# Hybrid 1D CNN-BiLSTM with focal loss for robust intrusion detection in in-vehicle CAN networks

**DOI:** 10.1371/journal.pone.0353472

**Published:** 2026-07-17

**Authors:** A. Malini, Gerardine Immaculate Mary

**Affiliations:** School of Electronics Engineering, Vellore Institute of Technology, Vellore, Tamilnadu, India; National University of Singapore, SINGAPORE

## Abstract

The Controller Area Network (CAN) bus serves as the primary communication framework in modern vehicles, enabling reliable data exchange among Electronic Control Units (ECUs) for seamless interaction. The CAN protocol lacks built-in protection, leaving in-vehicle networks vulnerable to cyberattacks such as denial-of-service, message injection, replay, and suppression.To address these challenges, this study presents a deep learning–based intrusion detection approach that integrates a 1D Convolutional Neural Network (1D CNN) with a Bidirectional Long Short-Term Memory (BiLSTM) model, along with focal loss to improve detection performance. The 1D CNN is responsible for capturing spatial patterns from CAN frame attributes, including arbitration ID, data length code, and payload information. In contrast, the BiLSTM learns temporal dependencies by analyzing sequence patterns in both forward and backward directions. Furthermore, instead of relying on synthetic data balancing techniques such as SMOTE, focal loss is applied to give greater importance to difficult-to-detect attack samples during training, thereby effectively addressing the issue of class imbalance in CAN datasets. Additionally window labeling tactic that helps detect attacks more sensitively within a temporal window is also proposed. Besides these, the Car-Hacking dataset (driving scenario) with stratified 10-fold cross-validation is used to test the above framework. The experimental results demonstrate that the proposed approach can achieve an accuracy of 97.41%, precision of 96.37%, recall of 96.73%, F1-score of 96.49%, and ROC-AUC of 0.9971 which is way above the baseline BiLSTM model performance from the previous work. These findings confirm that a combination of spatial feature extraction, temporal sequence modeling, and imbalance-aware optimization not only ensures but also delivers a highly effective and more reliable intrusion detection solution for in-vehicle CAN network security.

## Introduction

With the advancement of connected and autonomous vehicles (CAVs), the Controller Area Network (CAN) bus has emerged as a key communication platform for data exchange among Electronic Control Units (ECUs). Due to its low implementation cost, CAN-based networks offer high real-time performance along with reliable communication, making them widely adopted in modern vehicles. Therefore, CAN is nowadays extensively employed in various fields such as distributed process control systems, automated manufacturing, and in-vehicle communication [1]. Bosch developed CAN in 1985 as a way to reduce the car’s weight by using fewer wires. By means of CANs, a single network can connect multiple ECUs, which thus leads to a reduction in weight, an improvement of the network architecture, and control [2]. The massive use of entertainment equipment along with network topologies featuring more direct connections has, in the last couple of years, opened the door to new cyberattack channels [3]. Hackers can pose security and safety hazards for the driver and those around by exploiting vulnerabilities in the infotainment systems and the CAN, the primary communication protocol of in-vehicle networks. These breakings do not necessarily require the presence of the attacker at the physical access points [4]. By gaining access to the CAN bus, an attacker can carry out various types of attacks. Some of the best researchers in the world have documented comprehensive lists of attack scenarios [5,6] among which DoS, injection, replay, and suppression attacks are recognized as the most common ones. By means of the vehicle bus, intruders are enabled to infiltrate the vehicle’s internal network behind the user’s back and execute dangerous operations such as sudden acceleration or deceleration, the engine stopping altogether, emergency braking, or disabling the brakes, with all potential consequences of multi-layered security issues [7]. Moreover, in-car networks make the ideal target for cybercriminals. Since cyberattacks are capable of bringing about disastrous consequences in a short time, automakers highly need to consider equipping their future car models with well-designed and properly implemented security measures. Nevertheless, the CAN protocol has become very popular and has allowed manufacturers to produce mechanically more attractive and functionally expanded vehicles with a manageable number of wires. However, it still lacks built-in security features addressing the threat of hacking. Therefore, CAN messages in fact are “unprotected” information exposed to the whole network, and any compromised ECU is able to send the wrong messages to other ECUs without any checking. As a result, the absence of any security features of CAN naturally makes the CAN bus very exposed to attacks intended to exploit the faults of the system message spoofing, among others. The authors of these attacks can change the vehicle behavior and endanger passenger safety and vehicle reliability. For these reasons and at least for this reason security of the in-vehicle communication system is nowadays one of the hot topics in ITS research. Reliable CAN communication plays a crucial role in the operation of modern vehicle control systems. Interruptions, delays, or unauthorized modifications of CAN messages can adversely affect functions such as steering control, vehicle stability, and driver-assistance systems [8,9].Therefore, protecting CAN communications is important not only from a cybersecurity perspective but also for ensuring the safe and dependable operation of connected and autonomous vehicles. Various approaches and technologies have been utilized to address the above-mentioned security issues and vulnerabilities of the CAN protocol, including cryptographic verification mechanisms, intrusion detection (IDS) or anomaly detection (AD) at the network level aiming at identifying unauthorized use or anomalies in the network. Among these, IDS-based methods are considered to be the ECUs’ behavior monitoring tool as they can spot the abnormal communication pattern or the syntactic/semantic call to the service even when the CAN protocol is not modified. Recently, machine learning and deep learning techniques have been rapidly gaining ground in malicious CAN message detection due to their capability to analyze complex traffic patterns and thus determine the attack behavior. Specifically, experiments with deep neural networks such as convolutional neural network (CNN) or a recurrent neural networks (RNN) have been very successful in recognizing spatial as well as temporal features from the CAN traffic data. However, there still exist a few issues impeding the performance of an IDS applicable for in-vehicle use from its CAN platform. Actually, a CAN dataset can be significantly skewed as the normal message instances far exceed the attack message ones; therefore, finding a solution that would be accurate enough in attack detection is a challenge. Another problem is that a great number of existing methods deal only with extracting spatial or temporal features and thereby do not fully capture the nature of traffic. Finally, the variation in effectiveness of detection can depend a lot on how the labels are set for traffic windows; this is particularly the case during dynamic driving, in which the attack messages may be scattered here and there inside one sequence. Deep learning-based intrusion detection systems have significantly reduced the number of limitations, but several remain. For one, most existing CAN IDS approaches concentrate only on spatial feature extraction or temporal dependency modeling. So, they are partially characterizing the behavior of CAN traffic. Besides, the large imbalance of normal and attack messages usually makes classical loss functions bias the learning towards the majority classes. As a result, the detection of attack effectiveness decreases. Likewise, the question of how different sequence labeling strategies can affect the intrusion detection performance has not been thoroughly investigated so far, especially in the case of dynamic driving environments, where the attacking frames may be very few and far apart within the temporal sequence. Consequently, these difficulties call for the creation of a new intrusion detection framework that can simultaneously learn the spatio-temporal features, deal with the class imbalance, and enhance the attack sensitivity at the sequence level. To address these challenges, this study introduces a hybrid deep learning–based intrusion detection framework that integrates a 1D Convolutional Neural Network (1D-CNN) with a Bidirectional Long Short-Term Memory (BiLSTM) model. The 1D-CNN is used to learn spatial patterns from CAN data, whereas the BiLSTM captures temporal relationships by analyzing sequence information in both forward and backward directions. This combined architecture enables effective modeling of CAN traffic behavior. The key contributions of this work are summarized as follows:

- A hybrid 1D-CNN–BiLSTM intrusion detection framework is developed to learn both spatial and temporal characteristics of CAN traffic. The model incorporates focal loss to reduce the impact of class imbalance and employs sequence-level attack labeling to improve the detection of malicious activities within traffic windows.
- A Sequence-based labeling approach is employed in which a window is assigned an attack label if it contains at least one malicious CAN message. This approach increases the likelihood of detecting attack events occurring within a sequence and helps identify short attack bursts that may not be captured when labels are assigned solely based on the final message in the window.
- We proposed a hybrid deep learning framework by integrating 1D Convolutional Neural Networks and a stacked two-layer Bidirectional LSTM. The 1D-CNN layer identifies local byte-level spatial correlations in CAN payloads. Going further, backward and forward in time, the stacked BiLSTM layer captures temporal relationships very well. By combining both at different levels, not only a thorough grasp of driving behavior patterns is obtained but also a stronger representation of discriminative features which is basic to identifying anomalies.
- In order to raise the safety level even higher, a recall-driven threshold tuning technique is incorporated into each cross-validation fold. This step is directed at a minimum of false negatives, being the challenging condition in the automotive security domain.
- The proposed method is thoroughly validated through a stratified 10-fold cross-validation technique on the driving portion of the Car-Hacking 2020 dataset. The experimental results show that the proposed method not only has a great achievement in recall, F1-score, and PR-AUC but also is consistent when compared to the baseline LSTM-based intrusion detection methods.

The rest of the paper is structured as follows. Related Work section is devoted to the literature on intrusion detection methods for in-vehicle networks while mainly focusing on CAN bus security. Dataset Overview section describes the Car-Hacking dataset as well as the preprocessing steps for preparing the data for model training. Performance Evaluation and Architectural Impact Analysis section present the overall performance evaluation of the proposed model and also in detail the evaluation based on the architectural elements used. Impact of Window Labeling Strategy section analyses the effects of different window labeling methods on the intrusion detection performance. Impact of Focal Loss Parameter section examines the changes in model performance as a result of different values of the focal loss α parameter. Comparison with Baseline Model section shows and discusses the distinctions between the proposed approach and the BiLSTM-based method introduced in the baseline paper. Last but not least, Conclusion section summarizes the main findings of this study and points out potential directions for further research.

## Related Work

### CNN-based CAN IDS

To help protect a vehicle’s CAN bus, the study proposes an intrusion detection system (IDS) that leverages a deep convolutional neural network (DCNN). DCNN detects the malicious traffic and learns the network traffic patterns without manually crafted features [10]. A CNN-based intrusion detection system has been proposed by the authors to improve the CAN bus system’s security. To test the method, the authors created custom datasets by using the three car models. The experimental findings confirm that the proposed model can efficiently recognize the wired attacks on the CAN bus system with excellent performance [11]. The parameterized Relu helps temporal convolutional network to better learn. A paper provides an IDS based on CNN to protect the CAN bus system [12]. One research proposes a Temporal Convolutional Network-Based Intrusion Detection System (TCNIDS) to detect in-vehicle CAN network intrusion. The system uses natural language sequences of word vectors and CAN IDs to reduce the data size. The model leverages parameterized Relu to improve temporal convolutional network learning [13] CAN networks have a Recurrence Plot-based CNN (Rec-CNN) intrusion detection system, which is presented in the literature. The method converts the encoded arbitration IDs to recurrence images in order to capture temporal dependencies in CAN message sequences, which conventional methods typically ignore. The public dataset used to evaluate the model contains DoS, fuzzy, and spoofing attacks, and besides that, it is tested on a target vehicle with simulated attacks, showing excellent detection performance [14]. This study presents a convolutional neural network (CNN)-based intrusion detection system for CAN bus networks. Meanwhile, the U-CAN model is introduced as a segmentation-based approach that utilizes saliency detection along with Hamming distance techniques to analyze CAN traffic data. With an F1 Score of 0.997, U-CAN has been evaluated on both raw and reverse-engineered CAN frames, and thus, it can detect DoS, fuzzy, spoofing gear, and spoofing RPM attacks [15]. Federated Convolutional Neural Networks (CNNs) prove that a practicable privacy-preserving IDS (ImageFed) technique is feasible. To test the resilience of ImageFed in real-world scenarios, consider two situations that might hinder the performance of FL: clients that are non-independent and non-iid) and no training data available throughout the process. The resulting study shows that ImageFed is stable while achieving an average f1-score of 99.54%, an accuracy of 99.87%, and a low detection latency [16]. The proposed method converts CAN transmission data into CAN images that visually illustrate the assaults and therefore, they are detectable. The abnormalities can be detected by a light-weight CanNet image classification network. Data correlation is enhanced by the changed CAN image colour coding schemes and copula entropy. YFSonata traffic data helps to assure the effectiveness [17]. To secure in-vehicle CAN networks, a cross-layer intrusion detection system based on multi-task learning was proposed. This method not only uses features of CAN messages but also takes advantage of voltage-based physical layer fingerprints, a CNN-based classifier is able to simultaneously detect attacks and identify compromised ECUs. By coupling source recognition with behavior scrutiny, the technique smartly prevents impersonation and message tampering attacks, and at the same time it keeps detection latency low [18]. The system relies on spatiotemporal relationships to document messages, thus facilitating the model’s training and enhancing its accuracy. The tests show that with the help of the IDS, detection latency and memory consumption can be significantly reduced. In fact, it uses only 3.3% of the RAM required and works four times faster [19]. A novel CNN model (Ham-CNN) integrating Hamming distance as a new feature has been put forward. The research suggests using the Hamming distance between two consecutive packets as a signal for better detection efficiency. The Ham-CNN model demonstrates superior detection accuracy over the standard CNN algorithm [20]. An efficient lightweight multi-scale 1D-CNN has been launched for intrusion detection in CAN, which uses multiple depthwise separable Conv1D branches with different kernel sizes to extract diverse temporal fingerprint of attacks. A parameter-free softmax-based scale gate is utilized to perform adaptive feature weighting and as a result reduce computational complexity. The experiments on genuine CAN signal confirm a high detection performance accompanied by a low model complexity [21]. A CNN-based IDS, optimized through Flame Seeker Optimization, has been designed for IoMT setups. The approach leverages preprocessing and hyperparameter tuning to boost the performance, thus outclassing baseline machine learning algorithms in terms of accuracy and time on benchmark datasets [22].

### LSTM based CAN IDS

Desta et al. presented MLIDS, which is a CAN intrusion detection system singlehandedly using Long Short-Term Memory (LSTM) that can operate on raw complex feature space CAN data without the necessity of conducting system analysis on the data. The method uses separate LSTM models per arbitration ID and combines prediction errors into a single anomaly signal for window-based detection. Experiments on real and public data sets show that the method is effective for detecting insertion, fuzzy, DoS, and targeted attacks [23]. Hossain et al. have proposed an LSTM-based intrusion detection system (IDS) for in-vehicle CAN (Controller Area Network) to detect the DoS (Denial of Service), Fuzzing, and Spoofing attacks. The model captures temporal dependencies in CAN message sequences and is assessed on a real vehicle dataset as well as the Survival Analysis dataset. The experimental results show that the LSTM model obtains a high detection performance and even surpasses other conventional methods [24] The author has proposed a method called CANet which is an unsupervised deep learning-based intrusion detection system for the high-dimensional CAN bus data (Controller Area Network). The design uses two independent LSTM modules for each CAN ID to get the temporal dependencies, after which a fully connected autoencoder is applied to model the inter-signal relationships. The experimental results have shown that this approach can efficiently discover different attack varieties with a significant improvement true negative rate [25]. Yang et al. introduced the intrusion detection method based on Attention-LSTM neural network. LSTM incorporated the attention mechanism enables the model to assign adaptive weights to the most important features and at the same time enhance the sequential learning. When tested on the KDD-CUP99 dataset, the Attention-LSTM model showed better performance than CNN, RNN, and basic LSTM models in terms of detection accuracy and false alarm reduction [26]. This study proposes a novel approach to the design and evaluation of attack-tolerant anomaly detection algorithms. The work extends previous studies by relying on Long Short-Term Memory (LSTM) Networks for detecting anomalies in real-time. The algorithm has been trained to recognize abnormalities right away. Experiments conducted on actual vehicle data illustrate the results of the design process as well as those of the anomaly detection engine as a whole [27]. In order to detect anomalous behavior on a controller area network (CAN) bus resulting from tampering attacks, this paper comes up with the idea of using long short-term memory (LSTM) as a deep learning approach to anomaly detection. After going through five different loss functions, the LSTM method exhibits a higher detection rate and a smaller number of false positives [28]. This article message injection attack detection is a combination of Long Short-Term Memory-Recurrent Neural Network (RNN), cut-of value, and change transition detection, where directed graphs are used to represent message sequencing. It predicts malicious message injections into the CAN bus. The method reached an accuracy level of 97.32% while the detection time was only 2.5 milliseconds [29]. A network security proposal was made for an LSTM-based intrusion detection system where the main focus was PCA and Mutual Information for dimensionality reduction and feature extraction. After being tested on the KDD99 dataset, the PCA-powered LSTM model resulted in better binary and multi-class classification than the other models [30]. This article presents an automotive intrusion detection system that combines a lightweight GRU-based neural network and a low-complexity data representation method for real-time vehicle safety monitoring. It was shown that the system achieved a high classification performance and real-time performance in experiments using in-car embedded devices, and it may enhance the vehicle operating system’s intelligence [31]. This paper presents an enhanced VIDS to detect overlapped voltage attacks on in-vehicle CAN network. To separate overlapped voltage signals, an LSTM-based autoencoder is utilized, while keeping the unique fingerprint features of the original waveforms. The experimental results prove that the achieved VIDS can detect compromised ECUs that use distorted voltage patterns as an imitation, at an accuracy of 99.4%. While the attack success rate of the current VIDS methods decreases from 60–95% to 0–18% [32]. The CANival framework for multimodal analysis represents an optimization design of CANet. This approach unifies the statistical and deep learning techniques into one single model. A statistical method uses the Time Interval Likelihood model to monitor CAN signals with irregular timing in which the deviations of the expected time intervals bring about anomalies. Deep learning combines an LSTM network with an autoencoder model to analyses the abnormality of the CAN message data signal [33]. The author of the paper presents a powerful, novel, lightweight, vehicle-intrinsic, and DL-based IDS. The system fuses signature-based detection and anomaly-based detection methods. In the initial phase of signature-based detection, an ANN has been trained to detect the attacks that are well-known and have been used before. While in the second phase of anomaly detection, LSTM has been used to detect the new/unknown attacks by looking for anomalies in the data [34]. Ravi Kishore et al. have postulated a Bidirectional LSTM (B-LSTM) based intrusion detection framework for CAN bus networks. This framework officiates preprocessing techniques such as SMOTE-based class balancing, dropout regularization, and feature engineering for performance enhancement. Subjected to multiple CAN datasets from the Car Hacking Challenge, the B-LSTM model topped traditional ML methods and standard LSTM by exhibiting powerful anomaly detection capability [35]. In-vehicle Ethernet SOME/IP communication DDoS attack detection via a novel method that fuses Hawkes process with LSTM networks has been introduced. The Hawkes process characterizes the time varying and self-triggering nature of the attack events, whereas a specially designed HP-LSTM model with a residual attention mechanism is used for the detection task. The conducted experiments on a synthesized SOME/IP dataset show the promising performance of the proposed model in detecting DDoS attacks in the domain of connected and autonomous vehicles [36]. For the Internet of Vehicles (IoV), an intrusion detection system based on a CPSO-optimized Bidirectional LSTM (B-LSTM) network has been proposed to solve the problems of data imbalance and the detection of advanced attacks. The application of SMOTE is carried out for class balancing, whereas the use of Chaotic Particle Swarm Optimization (CPSO) is meant for hyperparameter optimization of the B-LSTM model. Results of the experiments on the Car Hacking Challenge 2020 dataset testify that the proposed method significantly outperforms LSTM and other optimization-based methods [37].

### CNN with BiLSTM based IDS

The hybrid deep learning-based IDS, HDL-IDS, specifically designed for in-vehicle networks, is introduced in this paper. HDL-IDS is a mixture of various techniques and methods from different domains, such as statistical analyses, knowledge graphs, and deep learning methods like Convolutional Neural Networks (CNN) and Long Short-Term Memory (LSTM) networks, to successfully classify network traffic and extract relevant features from raw network data [38]. The method is capable of detecting vehicle intrusions with a low false alarm rate and advanced attacks that demonstrate a clock skew in the real message. It is the first time an attempt has been made to detect frequency masquerade attacks by analyzing transmission time. The IDS efficiently detects malicious patterns by employing thresholds and attack statistical properties. It utilizes brute-force optimization for window sizes and involves a few legitimate data frames [39]. The technique proposed in this paper utilizes the inherent sequential nature of the CAN communication packets to achieve a decrease in detection latency and a reduction in energy consumption of certain systems.This paper aims to understand the response of NIDSs (Network Intrusion Detection Systems) based on deep learning with the use of adversarial inputs through TIKI-TAKA framework. Through its defensive/protection mechanism, this system is made tougher by the use of deception-based techniques. It has been found that attackers can identify 5 types of adversarial attacks and also can evade NIDS in 35.7% instances [40]. In order to detect network intrusions, a distributed, deep learning–based intrusion detection system is created on the Apache Spark platform. CNN and LSTM networks are used in this method to identify spatial as well as temporal patterns of vehicular CAN traffic, thus facilitating efficient anomaly detection. Results of experiments have shown that the system has better real-time performance and higher detection accuracy as compared to standard methods [41]. The idea of deep learning-based distributed intrusion detection system on Apache Spark platform has been proposed to detect the intrusions in IoV scenario. The system makes use of two types of neural networks, namely, CNN and LSTM networks, for the extraction of spatial and temporal features of network traffic so as to identify the anomalies. The results of the experiment indicate that there have been improvements in the real-time performance and the level of detection accuracy over the traditional methods [42] More so, a machine learning–based network intrusion detection system has been proposed which is based on the Recurrence Plot (RP) methodology, a technique that visualizes the CAN message bus communication. The visualizations not only represent the sequential characteristics of the messages but also the relationships within and across them. These images are subsequently used as a training dataset for a neural network that is designed for identification of the abnormal intrusions [43]. A driver categorization-focused IDS framework makes use of the LSTM-FCN architecture to obtain optimal strengths from the two network types, i.e., fully convolutional networks (FCN) and long short-term memory (LSTM) networks [44]. Besides that, the presented have facilitated superior outcomes as compared to other existing methods by reaching near perfect accuracies on both the Hacking and Countermeasure Research Lab (HCRL) dataset as well as test dataset. Furthermore, this work besides deep learning–based framework for improving Vehicle-Based Security System (VBS-D) mechanisms, it also enables the detection of physical intrusions even if malicious devices remain inactive. The approach was also put into practice through a CAN bus prototype where it kept the error levels very low and exhibited consistent reliable detection of intrusions under different operating conditions as well as temperature performance variations. In a live moving vehicle, with device identification accuracy reaching up to 99.8%, it demonstrates a high level of resilience [45]. Anitha et al. suggested a BI-LSTM–CNN deep learning framework for a smart device hostile traffic detection problem. This method uses word embeddings and extracts features from packets to understand both temporal and spatial relations in the network traffic. Evaluation on the ISCX2012 and USTC-TFC2016 datasets demonstrates that the proposed system attains a high detection rate while maintaining a low false alarm rate, thereby outperforming several existing deep learning approaches. correct data frames. Testing results show that the system has a low false-positive rate and its total error is reduced [46]. A part of the experiment was to check how well the proposed method works on a GPU-based automotive-grade embedded device. In order to detect anomalies in multi-sensor CAVs, the D-CNN LSTM Autoencoder architecture has incorporated two different data pre-processing methods and has been compared to deep learning-based models. The main novelty of this work is that it focuses on raising the quality of time series data using Differencing (DIFF) or Moving Standard Deviation (MSD). Besides, the D-CNN-LSTM Autoencoder model not only shows great performance in spotting high magnitude anomalies, but it also can boost the F1-score by 18.12% in the case of single types and by 32.83% in the case of mixed types when detecting low magnitude anomalies. The two-step method drastically improves the accuracy and robustness of the classification for the CAN bus network attacks and the F1-score attained was nearly 99.5% [47]. The Hybrid SecNet architecture consists of two classification phases: the initial one employs LSTM to decide whether the input is an attack or regular, and the subsequent one makes use of CNN to classify the different types of attacks [48]. They used a deep learning technique that involved Long Short-Term Memory (LSTM) networks united with an autoencoder design which has served to study the signals of the CAN messages and to perform anomaly detection. A gradient correlation-based linear method was used to indicate the presence of adversarial samples by a gradient correlation-based adversarial attack layer detection technique. And, thus, the technique boasts a improved performance accuracy rate. This method applies Gradient-Based Adversarial Algorithms. It is helpful for recognizing hostile attacks as well as for re-training the model with new data. Experimental samples have proved that this adversarial attack detection approach performs excellently with a detection accuracy of 99% [49]. Feature ranking, autoencoders, LSTM deep regressor, and fusion formed an object-oriented framework for intrusion detection and CAV mitigation.Three feature rankings—PCC, MRMR, and RReliefF—were employed to identify which overlapping heterogeneous sensors shared the least information with the target sensor. Detection and estimation phases were conducted with three 1-D CAEs and LSTMDR models, respectively, that were learned with the three identical redundant sensors. To further boost the effectiveness of intrusion detection, data fusion was also applied to resolve discrepancies between intrusion detectors [50]. In order to address the high false positive rates and restricted generalization of conventional deep learning techniques, a hybrid CNN–LSTM architecture has been suggested for network traffic anomaly detection. In order to detect complicated traffic patterns more effectively, the LSTM records temporal relationships while the CNN extracts spatial characteristics. When compared to solo CNN and LSTM models, experimental findings on the UNSW-NB15 dataset show better binary and multi-class classification performance [51]. As summarized in Table 1, previous studies on CAN intrusion detection have employed CNN-, LSTM-, BiLSTM-, and attention-based models to capture different characteristics of CAN traffic. CNN-based approaches are effective in extracting local feature patterns, whereas recurrent architectures are commonly used to learn temporal dependencies among CAN messages. Most existing methods, despite their potential, still depend on the traditional loss functions. Besides, they usually don’t take into account, in an explicit way, how class imbalance or the sequence level of a malicious example might affect the results. In order to address these issues, we decided to combine 1D-CNN and BiLSTM network with focal loss and attack-sensitive window labeling. This way, the model can not only learn the spatial and temporal features of CAN traffic but also get better in recognizing minority attacks and quick attack events.

**Table 1:** Comparison of architectural features of existing CAN intrusion detection methods and the proposed framework.

Method	CNN	LSTM/BiLSTM	Focal Loss	Attack-Sensitive Labeling	CAN IDS
LSTM IDS (2020)	×	✓	×	×	✓
BiLSTM IDS (2024)	×	✓	×	×	✓
ACL-IDS (2024)	✓	✓	×	×	✓
HybridSecNet (2024)	✓	✓	×	×	✓
Rec-CNN (2022)	✓	×	×	✓	✓
Proposed Method	✓	✓	✓	✓	✓

## Proposed method

**Fig 1:**
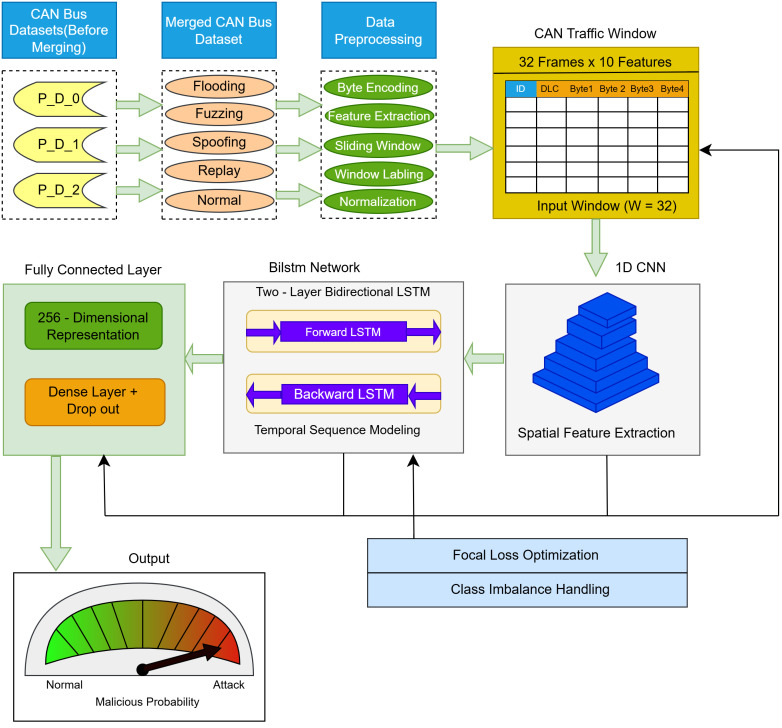
Proposed 1D CNN-BiLSTM intrusion detection framework for CAN bus networks.

The following section describes an intrusion detection framework that can detect anomalous or malicious behaviors even in the vehicle’s CAN network. The method here proposed performs spatial feature extraction through a 1D CNN and temporal sequence modeling by means of a deep BiLSTM network. Besides, a focal loss function is used to train the model so that the problem of class imbalance in CAN traffic datasets is better handled. The proposed framework example is given in Fig 1. The method consists of four components: CAN traffic preprocessing, sliding window construction, hybrid CNN–BiLSTM feature learning, and following classification. The configuration of the proposed model, including its architectural components and training settings, is presented in Table 2. The selected parameters were determined through experimental evaluation and based on configurations commonly used in deep learning-based intrusion detection studies.

**Table 2:** Hyperparameter settings of the proposed 1D CNN–Deep BiLSTM intrusion detection model.

Parameter	Value
Window Size	32
Window Stride	16
Input Features	10
Conv1 Filters	64
Conv1 Kernel Size	3
Conv1 Padding	1
Conv2 Filters	128
Conv2 Kernel Size	3
Conv2 Padding	1
Activation Function	ReLU
BiLSTM Layers	2
Hidden Units per Direction	128
BiLSTM Dropout	0.3
Fully Connected Dropout	0.4
Loss Function	Focal Loss
Focal Loss α	5
Focal Loss γ	2
Optimizer	Adam
Learning Rate	$1\times 10^{-4}$
Batch Size	256
Epochs	12
Cross-Validation	10-Fold

### Data preprocessing and window construction

A raw CAN dataset is essentially a file of timestamped messages, where each message contains several attributes such as the ID used for arbitration and the data length code (DLC), besides the original payload data bytes. Here, only a handful of the frame- level features from the arbitrated CAN message are chosen, namely, the ID of the arbitration, the length of the data, and the payload bytes. In order to make a machine-readable version of the payload data, it has been converted from hexadecimal to byte-level numerical values. So, each CAN frame can now be considered a 10-dimensional feature vector. Considering how CAN traffic passes through, the method allows the implementation of a sliding window segmentation approach. A sequence of 32 consecutive frames is what the system looks at to form an input with a fixed-length window size. Thus, each window is a 32 × 10 matrix, where 32 stands for the number of frames and 10 for the features extracted from each frame. Such a windowed representation lets the model figure out the sequence of communication patterns between the ECUs. Additionally, input data feature normalization is done to ensure consistency and a healthy training process. Let the CAN traffic dataset consist of a sequence of message frames,as expressed in (1):


$$\begin{array}{c} X=\{x_{1},x_{2},x_{3},\ldots ,x_{T}\} \end{array}$$ (1)

where $x_{t}\in {\mathbb{R}}^{10}$ represents the feature vector extracted from a CAN frame containing arbitration ID, DLC, and payload bytes.A sliding window of size Wis used to construct input sequence shown in (2).


$$\begin{array}{c} S_{i}=\{x_{i},x_{i+1},\ldots ,x_{i+W-1}\} \end{array}$$ (2)

where W = 32 in this study. Thus, each input sample can be represented as a matrix in (3).


$$\begin{array}{c} S_{i}\in {\mathbb{R}}^{W\times F} \end{array}$$ (3)

where F = 10 denotes the number of extracted features.

### Hybrid CNN-BiLSTM network architecture

To capture spatial and temporal properties from CAN traffic data efficiently, a hybrid deep learning model which that blends 1D CNN and BiLSTM is proposed. Primarily, a 1D CNN layer is leveraged to draw out spatial correlations between the frame-level features contained within each input window. Convolutional filters are working along the feature dimension to find local patterns in CAN messages such as the relationship between arbitration IDs, DLC values, and payload bytes. These spatial correlations are captured using a 1D convolution operation, which is defined in (4). The CNN module consists of two convolutional layers with 64 and 128 filters, respectively. These layers are followed by Batch Normalization and ReLU activation to ensure stable learning behavior and to introduce nonlinearity into the model.


$$\begin{array}{c} h_{j}=\sigma \left(\sum_{k=0}^{K-1}w_{k}\cdot x_{j+k}+b\right) \end{array}$$ (4)

where:

- $w_{k}$ denotes convolution filter weights
- $x_{j+k}$ represents the input value
- *k* indicates the kernel size
- *b* is the bias term
- α refers to the activation function (ReLU)

The extracted features are then provided to a Bidirectional LSTM network to model temporal relationships in both forward and backward directions. Unlike a conventional LSTM, the BiLSTM processes sequence data from two directions, allowing it to learn dependencies from both past and future CAN frames simultaneously. Each BiLSTM layer consists of 128 hidden units, resulting in a 256-dimensional feature representation after combining the forward and backward hidden states. The forward LSTM component is defined as shown in (5).


$$\begin{array}{c} {\vec{h}}_{t}=\text{LSTM}(x_{t},{\vec{h}}_{t-1}) \end{array}$$ (5)

The backward LSTM is defined as in (6):


$$\begin{array}{c} {\overset{\leftarrow }{h}}_{t}=\text{LSTM}(x_{t},{\overset{\leftarrow }{h}}_{t+1}) \end{array}$$ (6)

The final hidden representation is obtained by concatenating both directions in (7):


$$\begin{array}{c} h_{t}=[{\vec{h}}_{t};{\overset{\leftarrow }{h}}_{t}] \end{array}$$ (7)

where $h_{t}\in {\mathbb{R}}^{256}$. This dual spatial-temporal framework allows the model not only to learn the local feature interactions but also the sequential communication patterns from the CAN traffic, which, thus, greatly enhances the identification of network anomalies.

### Classification layer and output prediction

The final prediction is made by a fully connected (dense) layer receiving the high-level temporal features from the BiLSTM network. To avoid overfitting and thus enhance the generalization performance, a dropout layer was inserted between the dense layer and the input of this layer. The high-level feature vector is fed through a dense layer to compute the output logit, which is defined in a equation. Equation (8) shows the dense layer that converts the 256-dimensional feature vectors into a single neuron output.


$$\begin{array}{c} z=Wh+b \end{array}$$ (8)

where:

- $h\in {\mathbb{R}}^{256}$ denotes the feature vector.
- *W* represents the weight matrix
- *b* is the bias term

A sigmoid activation function defined in (3), is applied to the output neuron to generate a probability score representing the likelihood of malicious activity. The output value lies within the limit from 0 to 1, with values near 1 showing attack behavior and those close to 0 representing normal traffic. Based on a predetermined decision threshold, the system classifies each traffic window as normal or an attack. The sigmoid function in (9) produces the probability of malicious behavior as defined in (10).


$$\begin{array}{c} \sigma (z)=\frac{1}{1+e^{-z}} \end{array}$$ (9)

This produces the probability of malicious behavior:


$$\begin{array}{c} p=P(y=1|x) \end{array}$$ (10)

where *y* = 1 denotes attack traffic.

### Focal loss optimization for class imbalance

Invasion detection datasets for CAN most of the time suffer from class imbalance, where normal traffic is much more than the attack messages. The standard loss functions, such as binary cross-entropy, treat all samples uniformly, which may induce a model to be biased toward the predominant class. To address this class imbalance, the model is trained using Focal Loss, as expressed in (11):


$$\begin{array}{c} FL(p_{t})=-\alpha (1-p_{t}{)}^{\gamma }\log(p_{t}) \end{array}$$ (11)

where:

- $p_{t}$ denotes the predicted probability corresponding to the true class
- α represents the balancing parameter
- γ refers to the focusing parameter

In fact, the focal loss function becomes the loss function to be optimized. It addresses the problem by lowering the weight of well-classified examples and simultaneously increasing the importance of the difficult, misclassified ones, particularly those belonging to minority attack class [52]. Consequently, the method actually forces the model to focus more on identifying malicious actions on imbalanced datasets. Introducing focal loss, therefore, helps the proposed method to not only be more sensitive to attacks but also to keep a fairly balanced performance overall. The balancing parameter α and focusing parameter γ are the means of adjusting the degree to which minority class samples are considered important in training.

### Model configuration and parameter selection

In this paper, the hyperparameter values were fixed through preliminary testing and the consideration of the relationship between model complexity and detection effectiveness. A temporal window consisting of 32 consecutive CAN messages was selected for the representation of the one time interval data. A stride of 16 was further chosen to obtain a series of time windows partially overlapping with each other and thus preserving the continuity of the neighboring sequences. Overall, the arrangement provided adequate time context without significantly upping the computational time/cost. The module extracting features was made of two one-dimensional convolutional layers with 64 and 128 filters respectively. Such a setting allows the network to gradually learn more and more descriptive feature maps out of the given CAN sequences. We used the stacked BiLSTM model comprising 2 layers and 128 hidden units to model temporal dependences. To improve generalization and reduce overfitting, dropout was incorporated within the recurrent layers and before the final classification stage. Class imbalance was addressed using focal loss with parameter values of (α = 5) and (γ = 2). These settings were chosen after preliminary testing and provided stable training behavior together with improved recognition of attack samples. Similarly, instead of employing a fixed decision threshold, threshold selection was performed independently within each cross-validation fold by identifying the value that produced the highest F1-score. This procedure helped achieve a suitable balance between precision and recall during classification.

## Dataset overview

### Car – Hacking 2020 dataset

The experiments were set up to use the publicly accessible Car Hacking: Attack & Defense Challenge 2020 Dataset, which includes both normal and attack CAN messages that have been collected under realistic driving conditions. In this research, a publicly available CAN bus dataset was utilized and there were no human participants, animals or any personal identifiable data. Hence, obtaining ethics approval and informed consent was not necessary. Before implementing the suggested preprocessing method, every CAN message was encoded by arbitration ID,DLC, and eight payload bytes. The dataset contain two types of driving conditions:

- Stationary condition
- Driving condition

Because detecting intrusions in realistic dynamic conditions is more difficult, our work concentrates only on the driving condition. While driving, CAN traffic may change drastically over time with features such as an abrupt change in arbitration IDs, variations in the payload, and frequent control signal updates. In the stationary condition, the behaviour is comparatively stable and the messages are periodic. Therefore, in the driving condition, one can expect non-stationary message sequences and higher intra-class variation in normal traffic. Another important observation is that, legitimate driving actions such as pressing the gas pedal, braking, and making a steering adjustment might be mistaken as attack signs, thus increasing the overlap between normal and malicious patterns. Due to this increased behavioral complexity, intrusion detection is more difficult, and thus, spatial-temporal modeling has to be quite robust. Hence, the evaluation of the framework under the driving condition gives the detection performance a more realistic and safety-critical test. The driving condition data consists of three files corresponding to the normal driving behavior, and two files have four types of attacks. The attack files contain the following attacks: Spoofing, Fuzzy, Flooding, and Replay. These three separate files have been combined into a single dataset for a unified temporal modeling of the whole driving sequence.

### Data preprocessing

**Fig 2:**
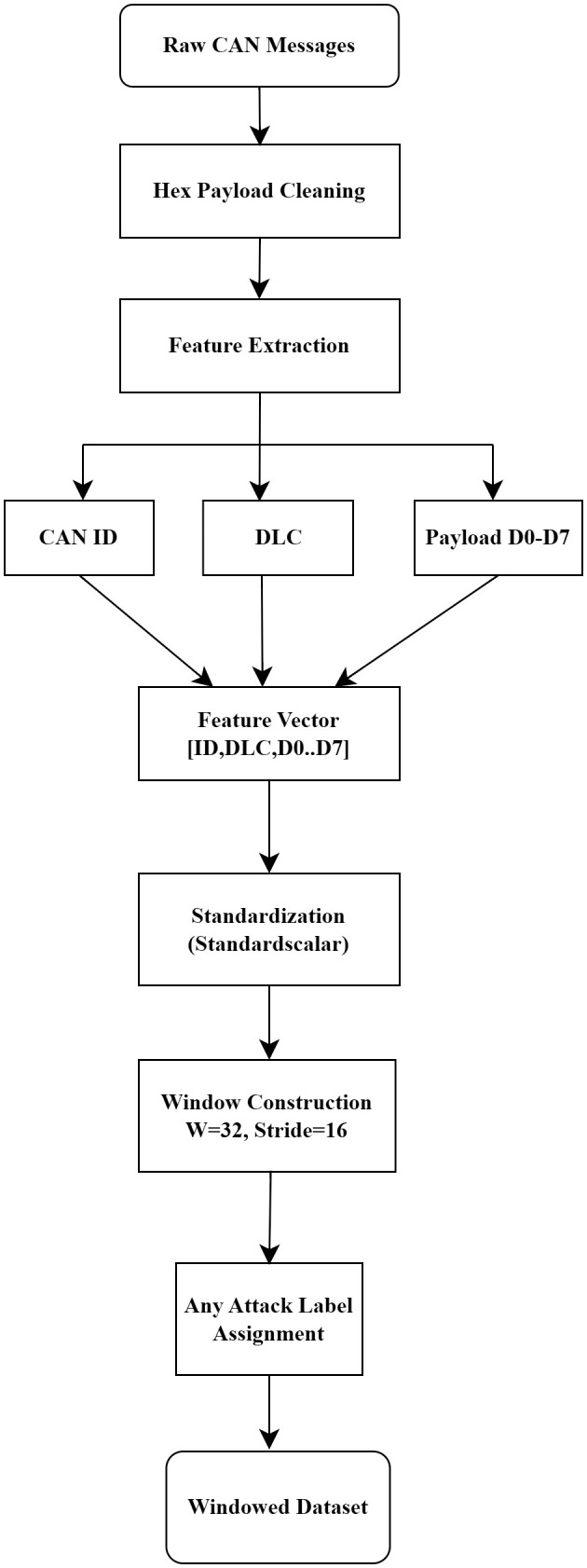
Data preprocessing workflow used for CAN intrusion detection, including feature extraction, normalization, window construction, and attack label assignment.

The raw CAN messages were first converted into numerical form before feeding them into the model for training. Arbitration identifiers were converted from hex to decimal values, while the DLC field was left as a numeric feature. Payload data were split across eight separate byte-level features (D0–D7), which gave the final feature vector for each CAN message consisting of the arbitration ID, DLC, and payload bytes. Class labels that were already given in the dataset were used to create binary targets where the normal traffic got a label of 0 and attacks were given a label of 1. Also, in order to catch the temporal dependencies among successive messages, windows that overlapped and contained 32 CAN frames were created with a stride of 16. A window was considered malicious when one or more attack messages appeared within the sequence; otherwise, it was treated as normal. Feature normalization was performed using StandardScaler to ensure that all input variables contributed on a comparable scale during training. To avoid data leakage, the normalization parameters were estimated from the training data of each cross-validation fold and then applied to the corresponding test data. Message timestamps were used only to maintain the original chronological order of CAN traffic during sequence generation and were not included among the input features. Fig 2 illustrates the sequence of preprocessing steps applied to the CAN traffic data, beginning with feature extraction and standardization, followed by sliding-window generation and window label assignment.

## Performance evaluation and architectural impact analysis

In order to thoroughly assess the impact of each architectural and optimization element, there are several setups were tested against each other using the same stratified 10-fold cross-validation experimental conditions:(*i*) GRU (*ii*) CNN (*iii*) BiLSTM with Binary Cross Entropy (BCE) loss (*iv*) BiLSTM with Focal Loss, and (*v*) the proposed model 1D CNN + Deep BiLSTM with Focal Loss method. The Table 3 briefly details the performance of each of the architectures tested in the paper. The GRU network scored a 90.67 ± 0.48% accuracy coupled with an F1-score of 86.25 ± 0.99%, suggesting that it could uncover connections across time in the sequences of CAN traffics. However, the CNN architecture answered with somewhat better statistics. In particular, it reached an accuracy of 91.93 ± 0.35% and an F1-score of 88.22 ± 0.78%. Even though these two models managed to pinpoint patterns of intrusion, their effectiveness was somewhat limited since they learned spatial and temporal information separately. The BiLSTM model with binary cross-entropy loss function averaged an accuracy of 93.58 ± 0.47%, precision of 93.26 ± 1.13%, recall of 88.86 ± 2.02%, and F1-score of 90.98 ± 0.78%, respectively. Besides that, the ROC-AUC and PR-AUC were 97.94 ± 0.24% and 97.27 ± 0.29%. Clearly, the BiLSTM is good at identifying the temporal dependencies in the CAN traffic. Nonetheless, the lower recall compared to other metric implies some vulnerability to the detection of certain attack windows. Switching the binary cross-entropy loss for focal loss up to a point enhanced the detection performance. Recall went up to 89.96 ± 0.74%, whereas F1-score climbed to 91.39 ± 0.42%. The ROC-AUC and PR-AUC also showed slight improvement with values of 98.21 ± 0.17% and 97.57 ± 0.21%, respectively. The model’s capability of recognizing rare classes, i.e., attacks, in an imbalanced dataset likely improved with focal loss that forces a more balanced attention to classes. Yet the improvement was minor overall pointing to the necessity of not only better feature extraction but also the first step in performance improvement. Among the different models tested, the hybrid 1D CNN–Deep BiLSTM design using the focal loss method produced the best results. This model was accurate by 97.41 ± 1.31%, precise by 96.37 ± 3.68%, recalled by 96.73 ± 0.89%, and F1-scored by 96.49 ± 1.59%. The pairs ROC-AUC and PR-AUC were also at impressive heights of 99.71 ± 0.05% and 99.56 ± 0.07%, respectively. The recall and F1-score improvements attest to the idea of bringing together the learning of spatial and temporal features in a single architecture as a winning one. The convolutional layers are responsible for discovering the local changes from the Arbitration ID, DLC, and payload bytes whereas the BiLSTM layers look at the ordered relationships among the different windows of messages. So, this mix of techniques gives an overall better characterization of the CAN traffic and leads to higher levels of attack detection. Generally, the evidences strongly point out the fact that focal loss together with CNN-driven feature extraction are the major factors that had contributed to the upgrading of the models performance. Their cohesive use in the authors framework has culminated in a more trustworthy intrusion detection model that is capable of dealing with the intricate attributes of CAN network traffic.

**Table 3:** Comparative performance analysis of deep learning architectures for CAN intrusion detection.

Model	Accuracy	Precision	Recall	F1	ROC	PR
GRU	90.67 ± 0.48	93.37 ± 3.07	80.36 ± 3.59	86.25 ± 0.99	96.38 ± 0.22	95.34 ± 0.26
CNN	91.93 ± 0.35	94.26 ± 1.95	83.01 ± 2.72	88.22 ± 0.78	97.42 ± 0.12	96.51 ± 0.16
BiLSTM (BCE Loss)	93.58 ± 0.47	93.26 ± 1.13	88.86 ± 2.02	90.98 ± 0.78	97.94 ± 0.24	97.27 ± 0.29
BiLSTM + Focal Loss	93.82 ± 0.32	92.89 ± 1.07	89.96 ± 0.74	91.39 ± 0.42	98.21 ± 0.17	97.57 ± 0.21
1D CNN + Deep BiLSTM + Focal Loss (Proposed)	97.41 ± 1.31	96.37 ± 3.68	96.73 ± 0.89	96.49 ± 1.59	99.71 ± 0.05	99.56 ± 0.07

To further examine the reliability of the results obtained, a Wilcoxon signed-rank test was conducted using the F1-scores of the ten cross-validation folds shown in Table 4. The proposed CNN–BiLSTM model was evaluated against the BiLSTM baseline as well as the BiLSTM model trained with focal loss. For both comparisons, the calculated p-value was 0.001953, which is lower than the commonly accepted significance level of 0.05. These findings indicate that the performance improvements achieved by the proposed framework are statistically significant and are not attributable to random fluctuations in the experimental results.

**Table 4:** Wilcoxon signed-rank test results using fold-wise F1-scores obtained from 10-fold cross-validation.

Comparison	Test	p-value	Significance
Proposed vs BiLSTM (BCE Loss)	Wilcoxon Signed-Rank	0.001953	Significant (*p* < 0.05)
Proposed vs BiLSTM + Focal Loss	Wilcoxon Signed-Rank	0.001953	Significant (*p* < 0.05)

### Results and discussion on architectures

**Fig 3:**
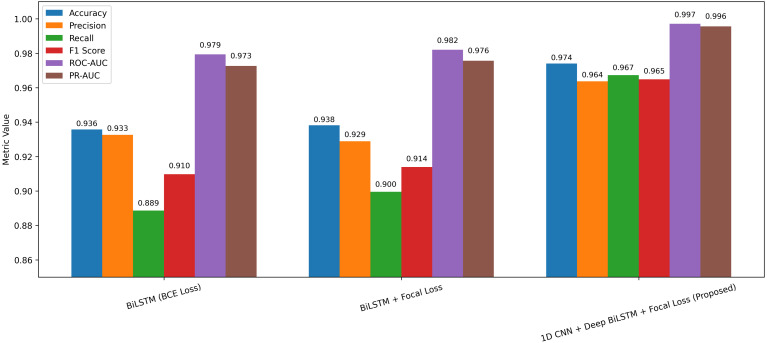
Performance comparison of intrusion detection models.

The experimental results reveal consistent performance growth between all sets of data, indicating that spatial representation, temporal modeling, and imbalance-aware learning should be optimized simultaneously. The baseline BiLSTM model has a high discrimination power; however, its lower recall indicates that it is susceptible to missing attack windows, which represents a very high risk in safety-critical vehicular environments. Using Focal Loss can only solve this problem to some extent by making the model put more emphasis on the hardest-to-classify attack samples. Changes in recall and F1-score demonstrate that imbalance-aware optimization is capable of minority class detection without the need for external data resampling or generation of synthetic samples. Still, the not very significant improvement shows that intrusiveness detection is mostly dictated by the effectiveness of the feature extractor. By using 1D CNN + Deep BiLSTM, the proposed new method for detecting cyber attacks achieved a high score in all the metrics, with the exception of precision, for which they scored a high rate of 0.96. Particularly, recall (96.73%) and F1-score (96.49%), which are very important to minimize false negatives in automotive security systems. By combining a spatial-temporal hybrid design, the model can depict not only the correlations of different modalities intra-frame but also their temporal dependencies inter-frame. This double-level feature learning makes it easier to differentiate between an attack and a valid change of control signals in a situation of a fast moving vehicle, and where the variations of the legitimate control signals are expected to be similar to those of malicious anomalies. A good ROC-AUC (0.9971) and PR-AUC (0.9956) demonstrate that the model’s ability to rank positive instances is very accurate and it is also capable of discriminating positive examples without depending on a threshold. Such a level of performance means that the proposed framework is able to manage a high safety level irrespective of the operating thresholds, making it compatible with real-life scenarios where detection sensitivity must be adjusted. In brief, the findings reconfirm that spatial-temporal feature integration leads to architectural excellence that results in a the major contribution to the performance of the model. Focal loss, on the other hand, is responsible for improving the sensitivity of the minority class. The comparative trends of the evaluation metrics across different architectures are further illustrated in Fig 3, where the proposed model consistently outperforms the baseline configurations in terms of precision, recall, and F1-score. Therefore, the introduced framework is a very effective and practically reliable method of intrusion detection for in-vehicle CAN networks operating under dynamic driving conditions.

## Impact of window labeling strategy

### Motivation for window-level labeling

Consider sequential CAN traffic frames as shown in (12)


$$\begin{array}{c} \{(x_{t},y_{t}){\}}_{t=1}^{T} \end{array}$$ (12)

where $x_{t}\in {\mathbb{R}}^{d}$ refers to the feature vector of the t-th frame and denotes its ground-truth label (0: normal, 1: attack). The intrusion detection mechanism presented in this paper functions on time series segments of length. Thus, a segment is defined in (13):


$$\begin{array}{c} X_{i}=\{x_{i},x_{i+1},\ldots ,x_{i+W-1}\} \end{array}$$ (13)

The technique of assigning a label to each segment has a direct effect on class distribution, severity of imbalance, and detection sensitivity.

### Labeling strategy definitions

Two methods for labeling windows were studied.

#### Last-frame labeling.

With last-frame labeling, the class of the window is determined by (14).


$$\begin{array}{c} Y_{i}=y_{i+W-1} \end{array}$$ (14)

thus only uses the last frame of the sequence to determine the window label. While this method is strictly temporal and sequentially consistent, it may disregard the cases of attacks that had appeared earlier in the window.

#### Any-attack labeling.

With any-attack labeling, the clss of window is determined by (15):


$$\begin{array}{c} Y_{i}=\left\{ \begin{array}{cc} 1, & \text{if }\exists \;t\in [i,i+W-1]\text{ such that }y_{t}=1\\ 0, & \text{otherwise} \end{array}\right. \end{array}$$ (15)

we can represent it as in (16):


$$\begin{array}{c} Y_{i}=\max(y_{i},y_{i+1},\ldots ,y_{i+W-1}) \end{array}$$ (16)

This method assigns a malicious label to the window if at least one of the frames within the window is an attack, thus increasing sensitivity to partial attack bursts.

### Impact on class distribution

The window labeling function $Y_{i}$ determines the distribution of the classes at the window level as in (17)


$$\begin{array}{c} D_{W}=\{(X_{i},Y_{i})\} \end{array}$$ (17)

Last-frame labeling results in a very imbalanced dataset:


$$\begin{array}{c} N_{\text{normal}}=107,790,\;N_{\text{attack}}=9,404 \end{array}$$

Any-attack labeling, on the contrary, leads to a more balanced distribution:


$$\begin{array}{c} N_{\text{normal}}=74,443,\;N_{\text{attack}}=42,751 \end{array}$$

Which in turn has reduced the severity of the imbalance and changed the model’s learning dynamics.

### Performance comparison

The effect of the labeling strategy can be seen in the evaluation metrics. Recall may be represented mathematically as in (20):


$$\begin{array}{c} \text{Recall}=\frac{TP}{TP+FN} \end{array}$$ (18)

**Fig 4:**
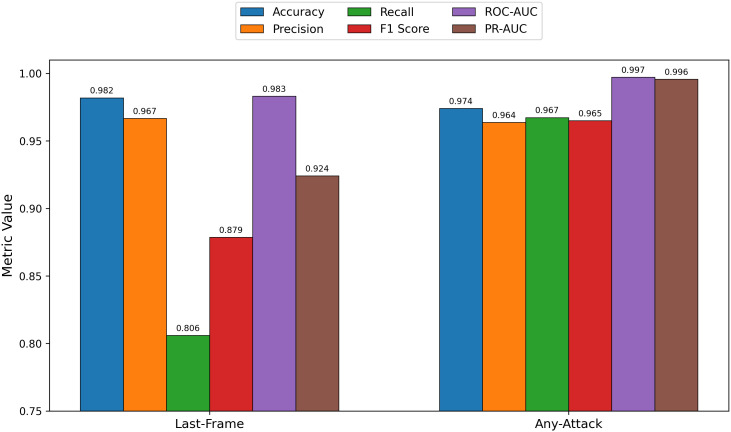
Performance improvement achieved using any-attack window labeling strategy.

The recall under the last-frame labeling method was 80.59%, and the recall with the any-attack labeling method was 96.73%. The corresponding F1-score went up from 87.87% to 96.49%, and PR-AUC went from 0.9241 to 0.9956. Even though the overall accuracy decreased somewhat due to a reduction in the bias towards the majority class, the significant decrease in false negatives is evidence of a better attack detection sensitivity. Fig 4 shows how the any-attack window labeling method improved performance. Table 5 displays the evaluation findings.

**Table 5:** Evaluation results of last-frame and any-attack window labeling methods.

Labeling Strategy	Accuracy	Precision	Recall	F1-Score	ROC-AUC	PR-AUC
Last-Frame	0.9822	0.9666	0.8059	0.8787	0.9831	0.9241
Any-Attack	0.9741	0.9637	0.9673	0.9649	0.9971	0.9956

### Practical implications for in-vehicle intrusion detection

In in-vehicle networks that are safety-critical, blast reduction is as important as detection. The any-attack labeling approach makes it possible for the intrusion detection system to identify malicious behavior quickly and reliably by detecting at least a part of the attack in the segment. That is why omitting the window labeling function from the discussion of the preprocessing stage is hardly justified, as it is a key component of the detection methodology influencing both the robustness and the practical feasibilty of the solution in the real world.

### Threshold sensitivity analysis

To assess how the decision threshold influences detection performance, the proposed model was evaluated at several threshold values, as shown in Table 6. At a threshold of 0.30, the model was able to recall (True positive rate) attack windows with a very high percentage of 97.68%, showing that almost all attack windows were caught. But then, at this threshold, precision was quite a lot lower. When the threshold was raised, precision increased gradually and hit the highest value of 99.48% at 0.70, while recall dropped to 89.59%. The results are a proof of the balancing act involved in detecting true attacks vs. generating false alarms. Using low threshold values results in a greater chance of detecting malicious activities but also increases the risk of misclassifying many normal windows as attacks. Conversely, higher thresholds will lower the number of false positives but the model will miss more attacks. The threshold picked after maximizing the F1-score gave the best trade-off and resulted in a powerful attack detection model along with a fair precision level that is suitable for automotive intrusion detection systems.

**Table 6:** Performance variation of the proposed model under different decision thresholds.

Threshold	Precision	Recall	F1-Score
0.30	0.9459	0.9768	0.9611
0.40	0.9676	0.9635	0.9655
0.50	0.9809	0.9469	0.9636
0.60	0.9880	0.9249	0.9554
0.70	0.9948	0.8959	0.9428

### Discussion on the any-attack labeling strategy

According to the proposed labeling technique, a window is labeled as an attack one if it contains at least one attack message. In this way, the model may be trained to recognize an attack which might happen only for a short time during the vehicle operation. In addition, the problem of missing momentary attacks is alleviated. Nevertheless, such a label has some shortcomings too. The attack label which is given to the entire sequence in a window may mislead the learning procedure if the number of malicious messages in a window is very low. In this case, the number of false alarms could rise. Another limitation is that since the output is at the window level, the model does not specify which message was the cause of the detection. This makes it hard to conduct a thorough analysis. However, for the cases of automotive security, the detection of malicious activities is considered an utmost priority since the consequences of successful attacks are the risk to human life. It is hoped that future work will come up with finer granular labels and message level detection algorithms that will make localized attacks more visible and still maintain effective detection.

## Impact of focal loss parameter

**Fig 5:**
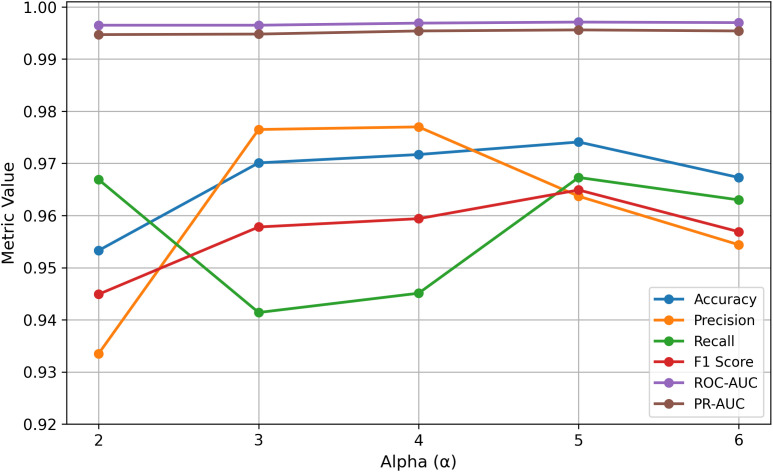
Effect of focal loss alpha parameter on IDS performance.

In order to understand how the class balancing factor in focal loss affects the model performance different values of α were tried from 2 to 6 without changing any other hyperparameters. The model was evaluated using 10-fold stratified cross-validation on the driving scenario of the Car-Hacking dataset. The performances obtained are reported in Table 7. The α parameter decides how many training samples of the minority class (attack windows) are considered in the model training. Lower values of α mean less emphasis on attack samples whereas higher values mean more weight to be assigned to misclassified attack instances. At α = 2, the model’s recall was really high at 0.9669, indicating that it was very sensitive to finding attack samples. However, the precision went down to 0.9335, meaning that there were more false positives produced. This is because with a lower balancing factor, the influence of easy samples is not sufficiently controlled, allowing the model to over-detect attack windows. Making α = 3 resulted in an even better harmony between precision and recall with an F1-score of 0.9578. The model didn’t lose any of the good attack detection features, and at the same time, it became more robust. At α = 4, the model went on to reach a new peak of F1-score of 0.9594 with precision of 0.9770 and recall of 0.9451. This combination offers an excellent balance between accuracy in detection and a lower number of false alarms. The best results were observed at α = = 5, where the model achieved the highest accuracy(0.9741), Precision(0.9637), recall(0.9673), and F1-score (0.9649) while maintaining balanced precision-recall performance. Hence, it can be concluded that the model is able to quite successfully handle hard attack samples without a significant rise in the false alarm rate. Nevertheless, performance went down a little at α = 6. Despite a still very good recall of 0.9630, the precision dropped to 0.9544, which caused the F1-score to go down as well. This means that if α is chosen to be too big, the attack samples might be over-prioritized, and therefore normal traffic can get wrongly classified as malicious. In summary, the main finding from the experiments is that the focal loss parameter α strongly affects the balance between precision and recall. The results suggest that α = 5 delivers the most balanced performance and consequently the highest overall detection ability for the CNN-BiLSTM network proposed here. As shown in Fig 5 increasing α from 2 to 5 gradually improves the detection capability of the model. Lower α values provide insufficient emphasis on minority attack samples, while excessively large values may over-penalize normal samples, resulting in increased false alarms. The best trade-off is observed at α = 5, where the model achieves the highest F1-score and balanced precision–recall performance.

**Table 7:** Performance comparison of different focal loss α values.

α	Accuracy	Precision	Recall	F1-Score	ROC-AUC	PR-AUC
2	0.9533	0.9335	0.9669	0.9449	0.9965	0.9947
3	0.9701	0.9765	0.9414	0.9578	0.9965	0.9948
4	0.9717	0.9770	0.9451	0.9594	0.9969	0.9954
5	0.9741	0.9637	0.9673	0.9649	0.9971	0.9956
6	0.9673	0.9544	0.9630	0.9569	0.9970	0.9954

## Comparison with the baseline model

**Fig 6:**
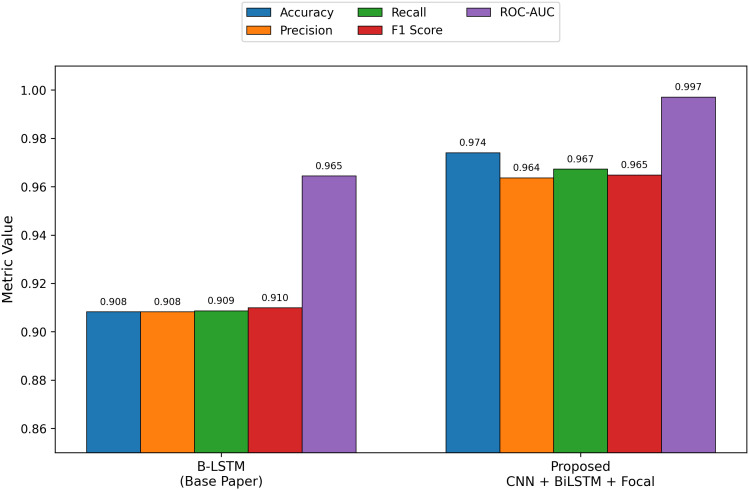
Performance comparison between the baseline BiLSTM model from the base paper and the proposed CNN–BiLSTM with focal loss model.

To strengthen the evidence of the proposed intrusion detection framework’s effectiveness, the experimental results of the proposed 1D CNN + BiLSTM + Focal Loss method are juxtaposed with those of the BiLSTM-based intrusion detection method from the baseline paper. The evaluation of both methods is done through widely accepted classificatory metrics, namely: accuracy, precision, recall, F1-score, and ROC-AUC.In the base article, the authors used SMOTE (Synthetic Minority Oversampling Technique) to render the dataset balanced prior to training the BiLSTM model. Although SMOTE resolves the problem of imbalanced data by creating new synthetic instances of the minority class, there is a risk of the generated data not being fully representative of real CAN traffic patterns. Different from that, the introduced method tackles the problem of imbalance from the perspective of model optimization by employing focal loss, a loss function that adaptively shifts the learning attention toward misclassified attack samples while lessening the impact of correctly classified normal samples. Thus, the model can raise the detection rate of the underrepresented class without altering the distribution of the original dataset. Fig 6 shows the comparative analysis between the suggested model and the baseline BiLSTM. The performance comparison is presented in Table 8. The BiLSTM baseline model documented in the primary work had an accuracy of 0.9083, precision of 0.9083, recall of 0.9087, and an F1 score of 0.9099, with an ROC AUC score of 0.9645. Despite producing a reasonable level of detection performance, the model’s effectiveness is very limited for sophisticated attacks concealed in the CAN traffic. On the other hand, the CNN + BiLSTM + Focal Loss method proposed in the paper produces the results very superior in all aspects with a value of 0.9741 for accuracy, 0.9637 for precision, 0.9673 for recall, and 0.9649 for the F1 score, as well as 0.9971 for ROC AUC. Judging by the metrics only, the model’s ability to detect attacks has been improved. It should be noted that only one driving scenario of the Car-Hacking dataset was used for the experiment on the proposed model and this scenario is more difficult than the stationary one since the CAN traffic is dynamic and changes a lot. Despite the fact that driving presents a more difficult scenario due to the continuously changing nature of CAN traffic, our method still manages to get a much higher F1 score than the baseline model. The results show that the combination of 1D CNN–based spatial feature learning and bidirectional temporal modeling with focal loss results in a more dependable and efficient intrusion detection system for vehicular networks. Overall, the comparative analysis confirms the advantages of the proposed method. In sum, the comparative analysis corroborates that the suggested method not only obviates the need for synthetic data balancing techniques like SMOTE but also realizes considerably enhanced detection performance under realistic driving circumstances.

**Table 8:** Performance comparison between the baseline BiLSTM model from the base paper and the proposed CNN–BiLSTM with focal loss model.

Model	Accuracy	Precision	Recall	F1-Score	ROC-AUC
Bi-LSTM	0.9083	0.9083	0.9087	0.9099	0.9645
Proposed CNN + BiLSTM + Focal loss	0.9741	0.9637	0.9673	0.9649	0.9971

## Conclusion

This study presents a reliable intrusion detection system for in-vehicle CAN networks based on a 1D CNN + BiLSTM hybrid architecture with focal loss optimization. The system is designed to proficiently understand the spatial correlations between the CAN frame features and the temporal relationships in the message sequences. Besides, focal loss mitigates the issue of class imbalance without the need for data resampling methods like SMOTE. Testing through the Car-Hacking dataset (driving scenario) with stratified 10-fold cross-validation reveals that the model developed herein leads to a considerable gain in performance over the BiLSTM baseline model. The system records an accuracy of 97.41%, precision of 96.37%, recall of 96.73%, F1-score of 96.49%, and ROC-AUC of 99.71%, which collectively imply that it is a highly accurate and dependable method of attack detection. The findings thus attest that a spatial–temporal deep learning with imbalance-aware optimization is a powerful and viable strategy to safeguard in-vehicle CAN networks.
